# Restricted Intimal Ca^2+^ Signaling Associated With Cardiovascular Disease

**DOI:** 10.3389/fphys.2022.848681

**Published:** 2022-03-22

**Authors:** Mark S. Taylor, Jordan Lowery, Chung-Sik Choi, Michael Francis

**Affiliations:** ^1^ Department of Physiology and Cell Biology, University of South Alabama College of Medicine, Mobile, AL, United States; ^2^ Department of Pathology, University of South Alabama College of Medicine, Mobile, AL, United States

**Keywords:** endothelium, atherosclerosis, artery, calcium, TRPV4 channels

## Abstract

Endothelial dysfunction is a key feature of cardiovascular disease (CVD) including atherosclerosis. Impaired endothelial signaling leads to plaque formation, vascular wall remodeling and widespread cardiovascular dysregulation. The specific changes along the vascular intima associated with atherosclerosis, including the vulnerable circulation downstream of the flow obstruction, remain poorly understood. Previous findings from animal models suggest that preservation of a distinct Ca^2+^ signaling profile along the arterial endothelial network is crucial for maintaining vasculature homeostasis and preventing arterial disease. Ca^2+^ signaling in the intact human artery intima has not been well characterized. Here, we employed confocal imaging and a custom analysis algorithm to assess the spatially and temporally dynamic Ca^2+^ signaling profiles of human peripheral arteries isolated from the amputated legs of patients with advanced CVD (peripheral artery disease and/or diabetes) or patients who had lost limbs due to non-cardiovascular trauma. In all tibial artery branches (0.5–5 mm diameter) assessed, the intima consistently elicited a broad range of basal Ca^2+^ signals ranging from isolated focal transients to broad waves. Arteries from patients with existing CVD displayed a restricted intimal Ca^2+^ signaling pattern characterized by diminished event amplitude and area. Stimulation of type-4 vanilloid transient receptor potential channels (TRPV4) amplified endothelial Ca^2+^ signals; however, these signals remained smaller and spatially confined in arteries from patients with CVD verses those without CVD. Our findings reveal a characteristic underlying basal Ca^2+^ signaling pattern within the intima of human peripheral arteries and suggest a distinct truncation of the inherent Ca^2+^ profile with CVD.

## Introduction

The vascular endothelium is a pivotal determinant of cardiovascular health. Risk factors including diabetes, hypertension and obesity along with altered shear stress along the vascular wall promote endothelial dysfunction (De Vriese et al., 2000; Barton, 2010). Characterized by impaired endothelium dependent vasodilation and increased oxidative stress, endothelial dysfunction is a harbinger of serious cardiovascular disease (CVD). Atherosclerosis is a common progressive disease process that involves deposition of cholesterol-rich plaque in affected conduit arteries and obstructive remodeling of the arterial wall, including inward growth of neointima into the vascular lumen (Gimbrone and García-Cardeña, 2016). Within major conduit arteries (e.g., carotid, coronary and arteries in the legs), narrowing of the lumen and thrombosis can severely compromise blood flow and ultimately cause life-threatening ischemia or loss of limbs. Indeed, coronary and peripheral artery disease (PAD) remain major health care challenges despite interventional strategies aimed at reducing risk factors, preventing thrombosis and reestablishing blood flow through angioplasty (Wolf and Hunziker, 2020). Moreover, vessels downstream of obstructive atheromas may be impacted by chronic low-flow inflammatory conditions. New strategies aimed at understanding and preserving endothelial function are warranted.

Maintaining normal Ca^2+^-dependent signaling along the endothelium is crucial for many processes that preserve vascular function, including nitric oxide signaling, secretion, and nuclear transcription (Falcone, 1995; Filippini et al., 2019). Disruption of these processes under inflammatory conditions such as diabetes is known to contribute to endothelial dysfunction and progressive vascular disease (De Vriese et al., 2000; Fitzgerald et al., 2007; Park et al., 2008). Specific changes in signaling along the vascular intima associated with atherosclerosis remain poorly understood. We and others have previously described distinctive profiles of basal dynamic Ca^2+^ signals in the arterial endothelium of rodents and pigs that occur even in the absence of exogenous stimulation and underpin inherent homeostatic Ca^2+^ signatures of the arterial intimal network (Ledoux et al., 2008; Sonkusare et al., 2012; Sullivan and Earley, 2013; Qian et al., 2014; Francis et al., 2016; Wilson et al., 2020). Within these signatures, discrete changes in the specific Ca^2+^ events (i.e., amplitude, duration and spatial spread or area) correspond to vascular function, including vasodilator and shear stress responses. Although effects of chronic pathologic signaling on endothelial Ca^2+^ signatures remain unknown, we previously reported that 2 weeks of reduced blood flow (in a partial ligation mouse model reducing carotid artery flow by >90%) substantially restricts the size and range of Ca^2+^ dynamics along the endothelial network (Taylor et al., 2017). One aspect of this adaptive shift may involve impaired expansion of locally triggered Ca^2+^ signals along the plasma membrane (e.g., Ca^2+^ sparklets occurring through type four vanilloid transient receptor potential channels, TRPV4). Indeed, TRPV4 channels have surfaced as particularly pivotal conduits for dynamic Ca^2+^ signaling in the endothelium (Bagher et al., 2012; Bubolz et al., 2012; Filosa et al., 2013; Liu et al., 2021) and altered TRPV4 signaling has been implicated in progressive cardiovascular disease (Nishijima et al., 2014; Zhang et al., 2018).

Dynamic Ca^2+^ signaling signatures along the intima of human arteries have not been studied. Here, we use confocal imaging and a custom analysis algorithm to characterize Ca^2+^ signaling profiles of peripheral arteries isolated from the amputated lower limbs of patients with or without existing CVD (peripheral artery disease/diabetes). We describe a robust dynamic Ca^2+^ signature along the intima of human arteries and a distinctive restriction of Ca^2+^ dynamics associated with existing CVD.

## Materials and Methods

### Procurement and Isolation of Artery Segments

Neurovascular bundle segments were removed from the limbs of patients who had undergone amputation surgery due to advanced cardiovascular disease or severe limb trauma. All tissues ware acquired after complete processing and evaluation by the University of South Alabama College of Medicine Department of Pathology. Patient gender, age, and the presence or absence of diagnosed peripheral artery disease and/or diabetes were recorded, and the tissue was deidentified. Tissues were stored in HEPES-buffered physiological saline solution (HBSS; in mM, 134 NaCl, 6 KCl, 1 MgCl_s_, two CaCl_2_, 10 HEPES, 10 glucose; pH 7.45) at 4°C and transported to the laboratory for study. Branches from the anterior and/or posterior tibial artery (three to five artery segments of 0.5–5 mm diameter) were dissected and employed in the experiments described within 8–18 h of amputation procedure. We have observed that extended tissue storage beyond this point can impact endothelial signaling and function. Notably, while most main tibial artery segments obtained from CVD patients contained extensive atheroma, the distal branches extracted for study did not have visible plaque and diameters were comparable among preparations employed. All arteries were treated identically, regardless of patient CVD status. Tissues were excluded from study if they were obtained beyond 18 h of the amputation surgery. All procedures were approved by the University of South Alabama Institutional Biosafety Committee.

### Ca^2+^ Imaging and Image Analysis

Artery segments were cut open longitudinally and mounted on sylgard inserts, intima side up, using tungsten micropins as previously described (Qian et al., 2013). When pinned, arteries are stretched to 1.5 times their resting width, approximating circumference at 80 mmHg as described previously (Francis et al., 2016). The tissue was then incubated with Ca^2+^ indicator loading solution containing Cal-520 AM (10 μM) at room temperature for 40 min in the dark. After washing and 30 min equilibration, inserts were inverted and placed in a glass-bottom chamber containing HBSS (tissue 100 μm from glass). The chamber was mounted on an Andor Revolution spinning disk confocal system (inverted microscope; ×20 objective, 0.75 NA), and Ca^2+^-dependent fluorescence was measured at eight frames/sec at 25°C using iQ software (1,024 × 1,024 pixels; 488 nm excitation, 510 nm emission). For each tissue sample, recordings were obtained from two separate intimal fields and subjected to analysis offline. 16-bit image sequence data were processed using a custom variation of the algorithm LC_Pro (Francis et al., 2012). This analysis software is specifically designed to: 1) detect sites of dynamic fluorescence change above statistical noise, 2) track signals continuously over x-y space and time, and 3) analyze fluorescence intensities within defined boundaries to determine specific event parameters. Here, we performed image-based signal detection in the python programming language. Briefly, input image sequences were noise filtered in time using the Savitzky-Golay algorithm and minimum background subtraction. Any image sequences displaying movement artifact were not included in analysis. Next, adaptive thresholding was applied via Gaussian convolution and the triangle thresholding method. Resultant binary masks were then generated, and object detection was performed using the scikit-image library. Objects corresponding to the calcium signal transients in each frame were then grouped into contiguous temporal events using a custom algorithm. For each signal event, fluorescence intensity, centroid, regional area and bounding box were measured for each frame. Fluorescence data are expressed as maximal amplitude (ΔF relative to a linear regression of background fluorescence), maximal duration (seconds), and maximal spatial area (µm^2^) for each event. A nuclear stain (NucBlue, Invitrogen; ex 405 nm, em 450 nm) was used to assess the status of the endothelial layer following Ca^2+^ measurements. Any areas lacking endothelial nuclei (identified as oval nuclei along the vessel surface) were circumscribed and assessed relative to the entire field using ImageJ; intimal fields with denuded areas >20% were not included in analysis.

### Immunofluorescence

Arteries were embedded in OCT and then cut into 20 µm cross-sections. After fixation in cold acetone for 10 min at room temperature, tissues were blocked in the blocking buffer (10% normal goat serum, 1% BSA in PBS) including 0.2% Triton X-100. TRPV4 protein was labeled with rabbit primary antibody (Alomone Labs) and Alexa 555 secondary antibody (Goat anti-Rabbit Alexa555; Invitrogen). Vascular endothelial cells were labeled with conjugated lectin-specific antibody (Tomato lectin-649; Vector lab). Elastic lamina autofluorescence was assessed using 488 nm excitation and nuclei were labeled with DAPI (405 nm excitation; Invitrogen). Digital image stacks were collected using a Nikon A1R confocal microscope (4X, 20X and 60X objectives) and NIS Elements software.

### Reagents and Solutions

Reagents were purchased from Sigma-Aldrich (St. Louis, MO), Cal-520 AM from AAT Bioquest (Sunnyvale, CA) and GSK1016790A Fisher Scientific (Pittsburgh, PA) Tungsten wires (for making pins) were purchased from Scientific Instrument Services (Ringoes, NJ).

### Data Analysis

Data are presented as means ± SE and/or as medians with full distributions where values are not normally distributed. All statistical analysis was performed with GraphPad Prism nine software. Due to limited tissue samples, data in respective groups were combined and analyzed as collective groups. Student’s t-test or Mann-Whitney was used for comparison of two independent Gaussian and non-Gaussian datasets, respectively. Kruskal–Wallis was employed for nonparametric analysis of multiple data sets with subsequent comparisons *via* Dunns post-test. *p* values <0.05 were considered significant.

## Results

### Patient Data

We obtained samples from 10 patients who had undergone elective amputation surgery due to either advanced clinical cardiovascular disease (PAD, diabetes or both) or non-cardiovascular related trauma. Two samples were excluded from study because they were obtained more than 18 h after the amputation procedure. One additional sample was excluded because the endothelium was severely damaged, precluding subsequent imaging and analysis. In all, samples from seven patients were used for study; four from patients who had diagnosed severe cardiovascular disease (CVD) and three from patients with no previously diagnosed cardiovascular disease (nCVD). Patient data is summarized in Table 1.

**TABLE 1:** Summary of patient data.

Patient	Gender	Age	PAD	Diabetes	Other notes
A	M	57	-	-	
B	F	71	-	-	
C	M	35	-	-	Fracture
D	M	60	+	-	
E	F	60	+	+	Renal disease + HTN
F	M	62	+	+	
G	F	59	+	+	Tobacco use

### Basal Dynamic Ca^2+^ Signal Profile Along the Intima of Peripheral Artery Branches

**FIGURE 1:**
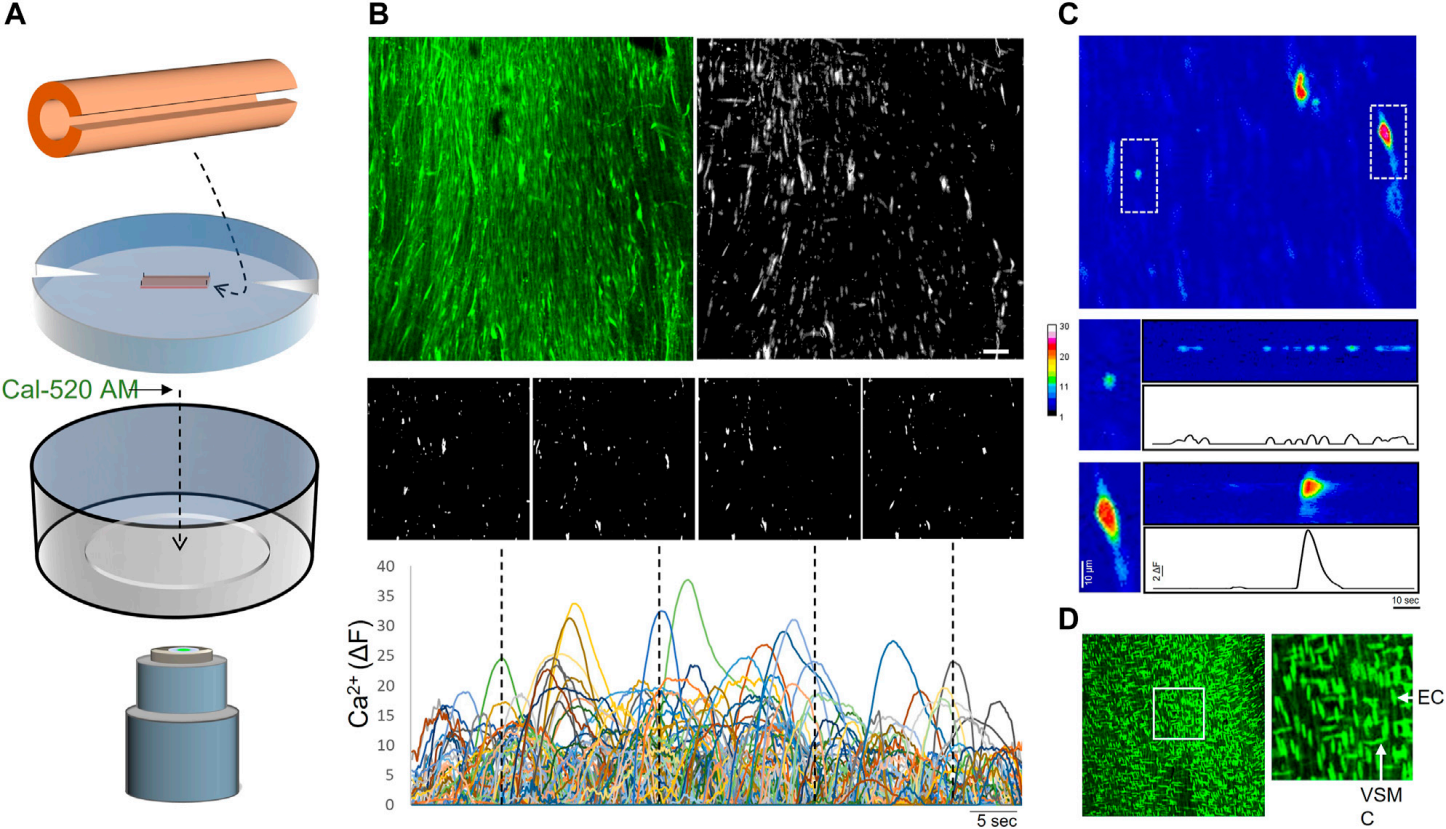
Imaging of intimal Ca^2+^ signals in open human artery segments **(A)** Opened artery segments mounted on silicone inserts were loaded with Ca^2+^ indicator (Cal-520 AM) and imaged using a spinning disk confocal microscope **(B)** Representative images (top) show maximal intensity projections (cumulative fluorescence over 60s) of an original recording and the Ca^2+^ signals detected along the vessel intima (monochromatic mask). A continuous time-lapse tracing was generated by tracking event centers over the sampling period; individual image panels show single-frame snapshots of Ca^2+^ signals at the time points indicated in the tracing (dotted lines) **(C)** A zoomed pseudo-color image in the sampled intimal field shows two distinct Ca^2+^ events (dotted boxes). The boxed regions (below) are compressed along the *y*-axis and extrapolated over time **(D)** Nuclear staining was performed after Ca^2+^ imaging to assess confluency of endothelial cells (vertically oriented nuclei) along the intima; image to the right shows boxed region. Bar is 50 μm; EC, endothelial cell; VSMC, vascular smooth muscle cell.

**FIGURE 2:**
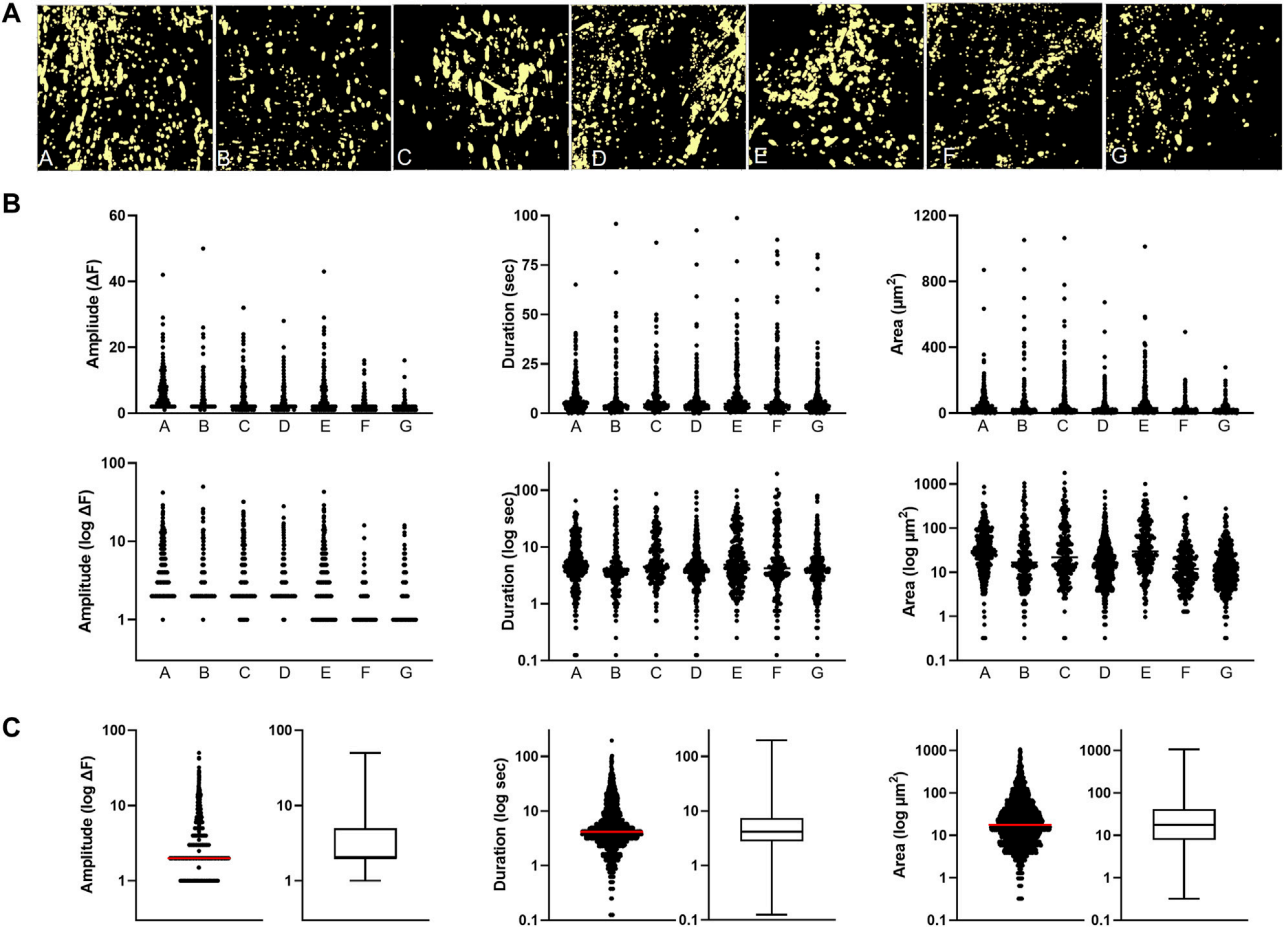
Basal dynamic Ca^2+^ signaling profile along the intima of peripheral artery branches **(A)** Cumulative masks of Ca^2+^ signals (yellow) obtained from different artery segments **(B)** Profiles of Ca^2+^ signal amplitude, duration and area are for the individual artery samples. Log transforms of the same data are also shown **(C)** Cumulative plots of all samples combined (median indicated by line).

In order to characterize the intrinsic dynamic Ca^2+^ signaling profile of the human peripheral artery intima, we performed confocal imaging on open artery segments loaded with the Ca^2+^ indicator Cal-520 AM (Figure 1A). Two-minute recordings revealed multiple Ca^2+^ transients occurring basally along the intima of all vessels assessed (Figure 1B). Heterogeneous Ca^2+^ signals were detected, ranging from focal repetitive transients to whole-cell waves (Figure 1C). All recordings were acquired from intact intimal fields exhibiting >80% endothelial cell coverage (Figure 1D). Figure 2A shows representative images of cumulative Ca^2+^ signaling patterns observed along the intima of different vessels. Overall, the frequency of events was 4.3 ± 0.7 s^−1^. Event parameters (amplitude, duration and area) were widely distributed and positively skewed in all vessels (Figure 2B). Mean amplitude, duration and area were 4.0 ± 0.1 ΔF, 7.6 ± 0.2 s, and 43.7 ± 1.9 µm^2^, respectively. Corresponding median values were 2.0, 4.1, and 17.4. Due to the substantial skew, data were log transformed for clarity (Figure 2B). Net parameter distributions are shown in Figure 2C.

### Distinctive Intimal Ca^2+^ Signaling Pattern Associated With Cardiovascular Disease

**FIGURE 3:**
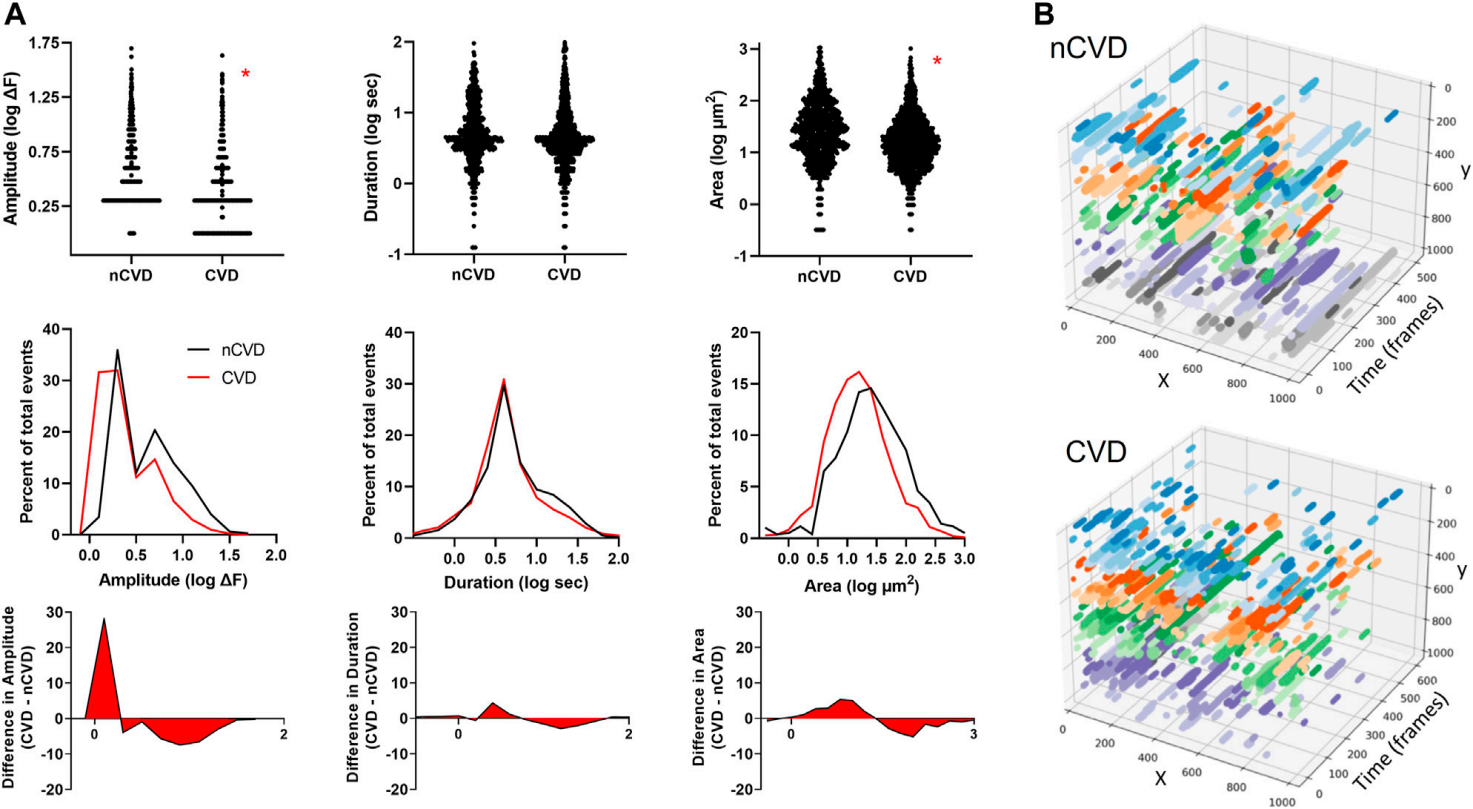
Distinctive intimal Ca^2+^ signaling pattern associated with atherosclerosis **(A)** Ca^2+^ signaling profiles of arteries from nCVD patients verses CVD patients. The middle panel shows overlapping distributions of parameters (amplitude, duration and area) and the bottom panel shows the difference between the CVD and nCVD distributions **(B)** Three-dimensional plots show x-y fields of Ca^2+^ signals projected over time for representative CVD and nCVD artery segments. Colors show relative event positions along the *y*-axis. Data were acquired from three nCVD and four CVD tissue samples. Asterisks (*) indicate *p* < 0.05.

Next, we compared the Ca^2+^ signaling patterns between arteries from CVD and nCVD patients. Overall, the frequency was 3.5 ± 0.8 s^−1^ (781 total events) in nCVD arteries compared to 4.9 ± 1.1 s^-1^ (1,446 total events) in CVD. This suggests higher event frequency in CVD arterial intima but the apparent difference did not reach statistical significance. Mean amplitudes were 5.5 ± 0.2 vs. 3.1 ± 0.1 ΔF (medians 3.0 vs. 2.0; *p* < 0.05), mean durations were 7.9 ± 0.3 vs. 7.4 ± 0.3 (medians 4.4 vs. 4.0), and mean areas were 62.5 ± 4.1 vs. 33.0 ± 1.7 (medians 26.4 vs. 14.8; *p* < 0.05). Figure 3A shows the relative event distributions in both groups; data are plotted as log values for clarity. In order to discern the specific differences in the event profiles, nCVD and CVD distributions were plotted as overlapping histograms. Subtraction of nCVD from CVD reveals the specific points of parameter deviation (peaks of red-filled plot) as well as the overall disparity in the distributions (area of red-filled plot). The data indicate a distinct trend for smaller Ca^2+^ events in CVD artery intima, especially with respect to amplitude and spatial area. The general truncation of event size is visually evident in the x-y-t plots shown in Figure 3B, where the recorded fields of Ca^2+^ signals are projected over time. The restricted stippled pattern in the CVD artery compared to the nCVD artery reflects the profile of smaller Ca^2+^ signals along the intima.

### Impact of TRPV4 Channel Activation on Dynamic Ca^2+^ Signaling

**FIGURE 4:**
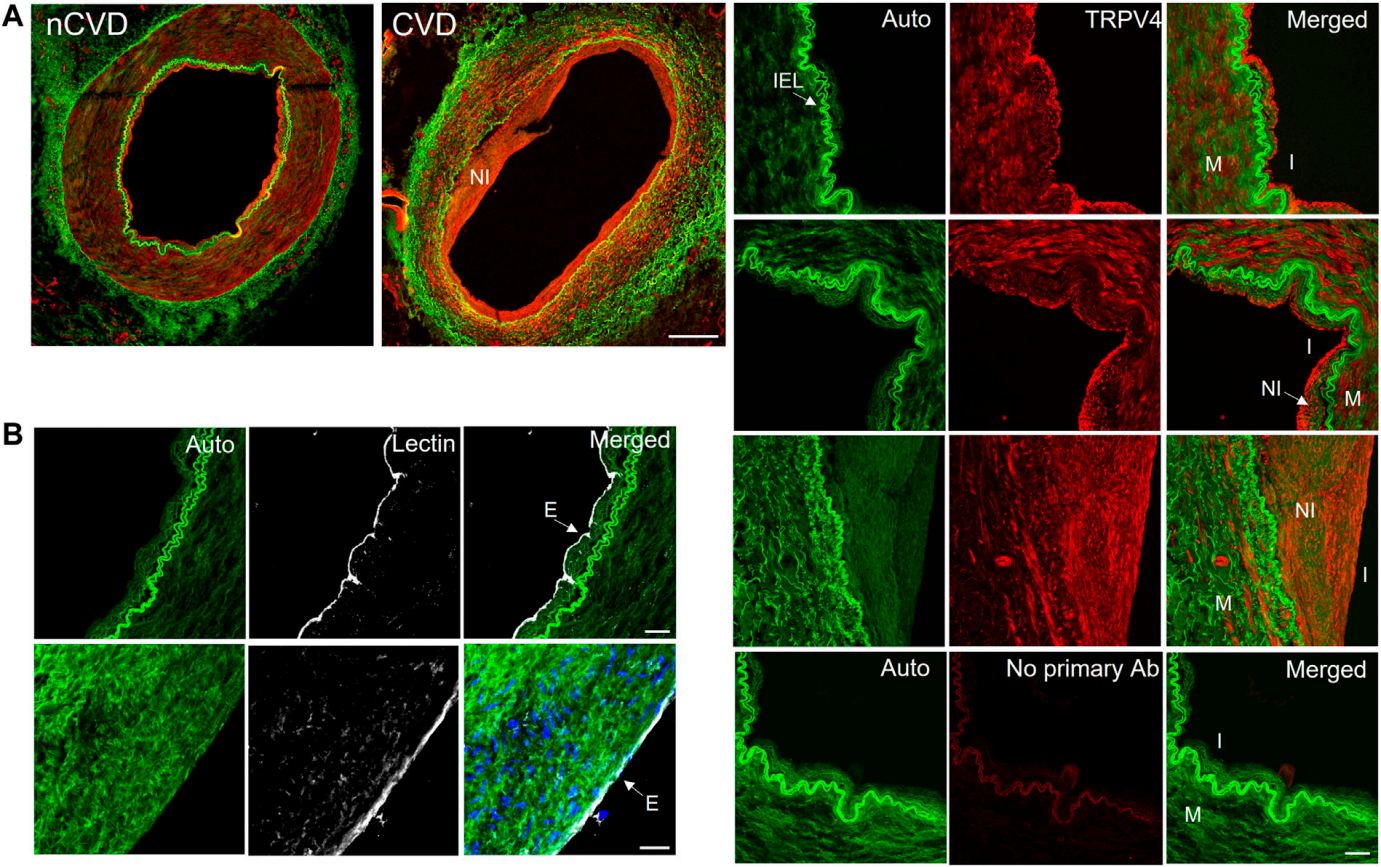
TRPV4 channel expression in human peripheral arteries **(A)** Immunofluorescence staining of TRRV4 (red) in cross-sections of nCVD and CVD peripheral arteries. Green is autofluorescence (Auto); Bar 500 µm. Panels to the right show autofluorescence (including internal elastic lamina; IEL), TRPV4, and merged signals. Images of the arterial walls of three different vessels show TRPV4 expression within the intima (I) and media (M). Expression was also detected in the neointima (NI) of all CVD arteries evaluated. Bottom panel shows secondary antibody-only control (No primary Ab); Bar is 50 µm **(B)** Lectin staining (white) showed consistent endothelial staining along the arterial intima, including vessels with developed neointima (cell nuclei indicated in blue); Bar is 20 µm. Data are representative from four separate samples.

**FIGURE 5:**
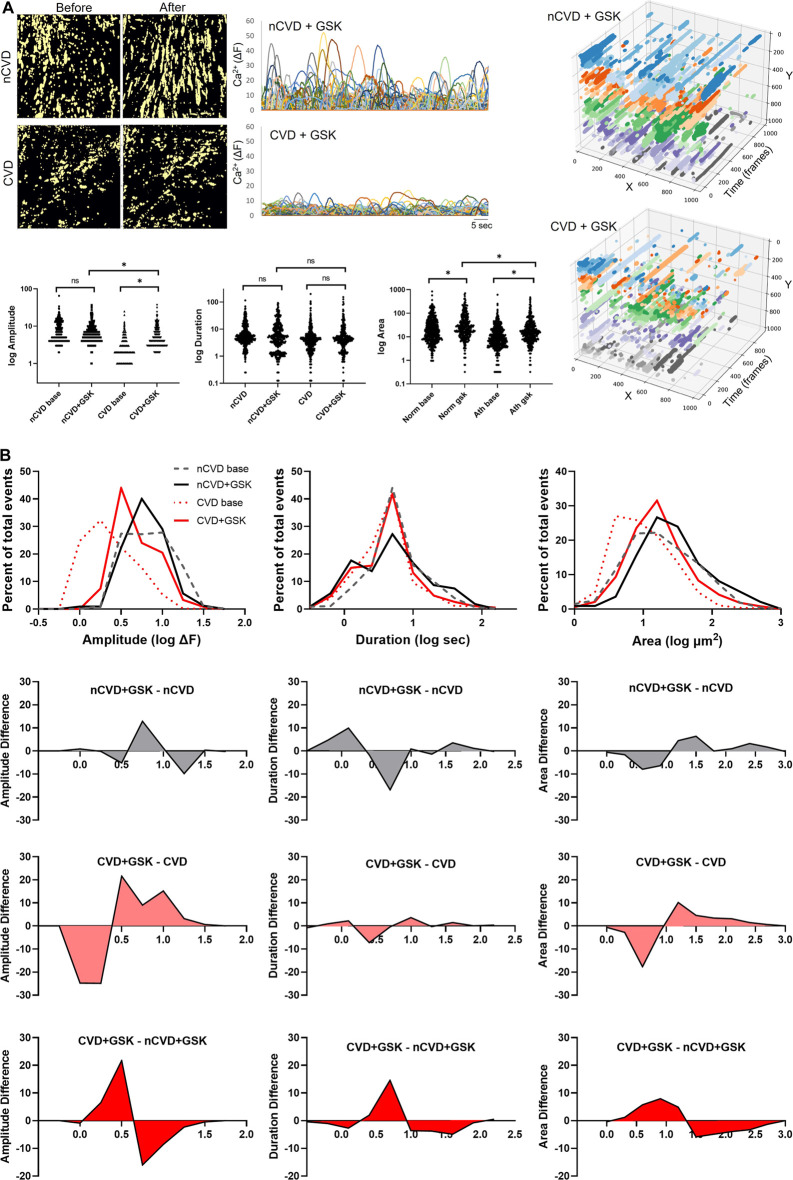
Impact of TRPV4 channel activation on dynamic Ca^2+^ signaling **(A)** Panels show cumulative (60 s) masks of nCVD and CVD arteries before and after 5-min treatment with 30 nM GSK1016790A. Tracings of individual tracked event centers are shown on the right and a summary of parameter distributions before and after GSK1016790A exposure is plotted below. Time-projected x-y plots of representative vessel preparations are shown on the right. Colors show relative event positions along the *y*-axis **(B)** Overlapping histograms of Ca^2+^ event parameter distributions and difference plots, showing the relative impact of GSK1016790A treatment and differences between CVD and nCVD arteries. Data were acquired from five different tissue samples. Asterisk * indicates *p* < 0.05.

TRPV4 channels are pivotal conduits for dynamic Ca^2+^ signaling in the arterial endothelium, and their capacity to generate and relay Ca^2+^ signals may be altered in cardiovascular disease. We extended our evaluations to assess the expression and relative effect of TRPV4 stimulation in CVD and nCVD arteries. Immunofluorescence staining (Figure 4A) of peripheral vessel cross-sections revealed consistent TRPV4 expression in the intimal and medial layers of both nCVD and CVD arteries. CVD arteries exhibited variable degrees of neointima, and TRPV4 expression was also evident within the neointimal layer. Notably, the endothelium was intact in all vessels assessed, including those with substantial neointima (Figure 4B). We assessed effects of TRPV4 stimulation on Ca^2+^ dynamics in a subset of arteries using the agonist GSK1016790A (Figure 5). Exposure to 30 nM GSK1016790A reduced the frequency of events in both nCVD and CVD arteries (from 4.6 ± 0.5 s^−1^ to 3.5 ± 0.2 s^−1^ in nCVD and from 5.5 ± 0.6 s^−1^ to 4.1 ± 0.06 s^−1^ in CVD), possibly reflecting the general coalescing of many local transients into continuous events and waves (Figure 5A). Overall, GSK1016790A exposure significantly increased the spatial area of nCVD events (36.3 ± 2.8 µm^2^ to 53.5 ± 4.7 µm^2^) and both the amplitude and area of CVD events (3.1 ± 0.1 ΔF to 5.8 ± 0.2 ΔF and 16.2 ± 1.3 µm^2^ to 30.7 ± 2.6 µm^2^, respectively); Exposure to vehicle (0.01% DMSO) had no effect. Both event amplitude and area remained lower in GSK1016790A-stimulated CVD arteries compared to stimulated nCVD arteries. The truncation of events in CVD vs. nCVD arteries is evident in the time-projected plots shown in Figure 5A. Overall, parameter distributions remain left-shifted in CVD arteries compared to nCVD arteries, supporting a general restriction of the Ca^2+^ signal profile (Figure 5B).

## Discussion

While distinct functional patterns of endothelial Ca^2+^ dynamics are beginning to emerge in various animal models and different vascular beds, the base Ca^2+^ signaling patterns of human arteries have not been characterized. The primary goal of the current work was to assess the prevailing intimal Ca^2+^ signaling signature in human arteries and provide insight on whether this signaling might be impacted by CVD. We analyzed Ca^2+^ signals along the intima of peripheral arteries isolated from the limbs of patients who had undergone amputation due to either severe cardiovascular disease or non-cardiovascular trauma. We identified a distinct dynamic Ca^2+^ signaling profile along the human arterial intima composed of a broad range of basal Ca^2+^ transients. This profile is notably muted in CVD arteries compared to nCVD arteries, with CVD arteries exhibiting a predominance of smaller focal events and fewer whole-cell waves. Stimulation of TRPV4 channels expanded intimal Ca^2+^ signals, particularly spatial area, in both CVD and nCVD arteries, but the overall signal profile remained reduced in CVD arteries. The current findings reveal an intrinsic Ca^2+^ signaling signature along the arterial intima of human arteries that is restricted in CVD.

Studies over the past decade have exposed the crucial impact of dynamic endothelial Ca^2+^ signals in tuning vascular responses including endothelium derived hyperpolarization and nitric oxide mediated vasodilation (Kansui et al., 2008; Ledoux et al., 2008; Qian et al., 2013; Francis et al., 2016). While endothelial dysfunction is known to play a crucial role in progressive CVD, particularly in the context of diabetes and atherosclerosis, recent findings indicate that early disruption of inherent signaling patterns along the endothelial network is pivotal in this transition (Taylor et al., 2017; Wilson et al., 2020). For instance, constrained or redirected Ca^2+^ signal communication may alter nitric oxide production and favor production of reactive oxygen species. The inherent signals underlying endothelial function in human arteries have not been well-studied due to limited tissue access and difficulties associated with measurement and quantification. Our lab and others have recently begun to apply imaging and analysis approaches to decipher complex dynamic signaling patterns in intact tissues. In the current study, we applied a next-generation version of our custom algorithm LC_Pro that has the distinct advantage of tracking events without applying fixed regions of interest (ROIs). This approach allows for detection and isolation of a wide range of events as well as the characterization of each event with space and time. Such continuous spatio-temporal tracking distinguishes events that might otherwise be coincidently sampled and misinterpreted by fixed ROIs, providing a valuable approach for future elucidation of signals within complex networks.

It is important to note that in the current study we only evaluated arteries with intact endothelia in order to capture continuous intimal Ca^2+^ profiles. This also ensured comparable readouts for CVD and nCVD vessels, allowing us to assess altered signaling in the diseased circulation, even where the intima remained intact. Indeed, we observed that while many arterial branches from CVD patients exhibited some degree of neointima (quite distal from the established upstream atheroma), the endothelium was usually intact. For Ca^2+^ signal quantification, we have found that tracking frequency as well as maximal amplitude, duration and area of detected events provides a useful assessment of signal profile. We noted a trend of increased event frequencies in CVD arteries although additional sampling in future studies will be needed to determine if this is a common feature of CVD or whether it may be specific to a condition, disease, age or some other variable. Analysis of Ca^2+^ parameter profiles showed that CVD artery distributions for amplitude and area were consistently left-shifted compared to nCVD arteries, suggesting the arterial intima of CVD patients elicit truncated Ca^2+^ signals compared to those of non-CVD patients. Overall, analysis of distribution differences revealed that CVD arteries have a higher density of events with amplitudes of one to three ΔF, durations of 2.5–5 s, and areas of 4–16 μm^2^, and a lower density of events with amplitudes of 5–10 ΔF, durations of 10–40 s, and areas of 30–250 μm^2^ compared to nCVD. This truncation of Ca^2+^ event size is similar to the effect of chronic low flow on mouse carotid arteries wherein 2 weeks of reduced blood flow resulted in endothelial dysfunction and significant arterial wall remodeling, including neointima formation (Taylor et al., 2017). We observed variable degrees of neointima in peripheral arteries from CVD patients in the current study. It will be useful in future studies to determine whether general endothelial dysfunction (impaired vasodilation) and remodeling of the peripheral vasculature (distal to obstructive atherosclerosis) correspond to the degree of endothelial Ca^2+^ signal disruption. In the current study, arteries from patients with severe PAD and/or diabetes were grouped into the CVD cohort. Extended studies should allow discrimination of functional Ca^2+^ signaling profiles between these groups and among other variables.

TRPV4 cation channels elicit focal Ca^2+^ influx events at the plasma membrane (Ca^2+^ sparklets), allowing for localized signal targeting and providing a trigger for larger Ca^2+^-induced Ca^2+^ release events (i.e., from the endoplasmic reticulum) (Sullivan et al., 2012; Lin et al., 2015; Heathcote et al., 2019; McFarland et al., 2020). Growing evidence supports involvement of these channels in shear stress and agonist-mediated endothelial signaling, and changes in TRPV4 signaling are linked to disease states including hypertension, diabetes and pulmonary edema (Hartmannsgruber et al., 2007; Mendoza et al., 2010; Sonkusare et al., 2012; Ma et al., 2013; Monaghan et al., 2015; Zhang et al., 2018; Daneva et al., 2021; Rajan et al., 2021). In the current study, we observed TRPV4 channels expressed in the intima as well as the media of peripheral arteries, and even within neointima of arteries from CVD patients. Our data suggest that stimulation of these channels expand Ca^2+^ signals along the intima, particularly the spatial area of events. However, while TRPV4 stimulation substantially increased the magnitude and reach of Ca^2+^ events in CVD arteries, these events remained diminished compared to nCVD arteries. This reduced signaling capacity in peripheral arteries of CVD patients suggests the channels generate initial Ca^2+^ transients but the amplification or spread these signals is impaired. We previously reported that spatially restricted endothelial Ca^2+^ events in mouse carotid arteries exposed to chronic low flow similarly failed to expand normally following acetylcholine stimulation. One possibility is that ion channels such as Ca^2+^-activated K^+^ channels that normally amplify these local signals and allow them to trigger internal Ca^2+^ store release are impaired or decoupled from the TRPV4 channels in the plasma membrane. Such decoupling of TRPV4 and K_Ca_2.3 channels has been implicated in vascular dysfunction associated with hypertension (Ma et al., 2013). The implication is that changes due to low flow and/or chronic inflammation reduce the capacity for expanding Ca^2+^ transients (e.g., Ca^2+^ sparklets). Extended investigation of the mechanism driving this shift and the implications for TRPV4 mediated signaling under conditions of shear stress in CVD is warranted.

The current findings underscore the complexity of biological Ca^2+^ profiles and the importance of discerning specific Ca^2+^ signal distributions. While mean parameter values can provide basic information about the events, assessment of full distributions is needed to characterize the disparate nature and range of signals. This will be particularly important for distinguishing Ca^2+^ parameter shifts with specific perturbations (e.g., receptor agonists, shear stress) and determining distinct physiologic and pathologic impacts. Indeed, our data suggest TRPV4 channel stimulation has a complex effect on the endothelial Ca^2+^ signal profile. While spatial area of the signals expands, as evidenced by a general right shift in the event area distribution, the profile for event duration appears to flatten, essentially redistributing to the left and right. This may reflect the widespread triggering of focal Ca^2+^ entry events and subsequent spread of many of these events as internal Ca^2+^ stores are recruited. Notably, this expanded duration profile is not evident in CVD arteries. A key point is that shifts in a parameter distribution may provide important information not reflected by mean values. Overall, our current findings suggest CVD limits the capacity of TRPV4 signaling. Specific aspects of these changes, including the mechanism underlying redirection of Ca^2+^ signals and the direct functional consequences remain to be determined.

It should be noted that cells other than endothelial cells were sometimes captured in our Ca^2+^ imaging studies. These are likely smooth muscle cells just below the internal elastic lamina or part of the neointima of CVD arteries. As these cells were part of the continuous cell population and we could not definitively identify and remove specific cells from analysis, they were included. Overall, we estimate these cells contributed <5% of the signals analyzed and had little impact on the net assessment. Future iterations of the imaging and analysis applied here should allow for discrete discrimination of the vascular intima as well as the specific impact of the neointima itself. This should also include assessment of endothelial status and the relative impact of apoptotic or necrotic cells on the prevailing Ca^2+^ profile. While the current study was limited by the modest number of tissue samples, the findings serve as a valuable foundation for future mechanistic investigations and development of new approaches for preserving homeostatic endothelial signaling.

## Data Availability

The original contributions presented in the study are included in the article. Further inquiries can be directed to the corresponding author.

## References

[B1] Bagher P. Beleznai T. Kansui Y. Mitchell R. Garland C. J. Dora K. A. (2012). Low Intravascular Pressure Activates Endothelial Cell TRPV4 Channels, Local Ca 2+ Events, and Ik Ca Channels, Reducing Arteriolar Tone. Proc. Natl. Acad. Sci. U.S.A. 109, 18174–18179. 10.1073/pnas.1211946109 23071308 PMC3497745

[B2] Barton M. (2010). Obesity and Aging: Determinants of Endothelial Cell Dysfunction and Atherosclerosis. Pflugers Arch. - Eur. J. Physiol. 460, 825–837. 10.1007/s00424-010-0860-y 20635093

[B3] Bubolz A. H. Mendoza S. A. Zheng X. Zinkevich N. S. Li R. Gutterman D. D. (2012). Activation of Endothelial TRPV4 Channels Mediates Flow-Induced Dilation in Human Coronary Arterioles: Role of Ca2+ Entry and Mitochondrial ROS Signaling. Am. J. Physiology-Heart Circulatory Physiol. 302, H634–H642. 10.1152/ajpheart.00717.2011 PMC3353785 22140047

[B4] Daneva Z. Marziano C. Ottolini M. Chen Y.-L. Baker T. M. Kuppusamy M. (2021). Caveolar Peroxynitrite Formation Impairs Endothelial TRPV4 Channels and Elevates Pulmonary Arterial Pressure in Pulmonary Hypertension. Proc. Natl. Acad. Sci. USA 118, e2023130118. 10.1073/pnas.2023130118 33879616 PMC8092599

[B5] De Vriese A. S. Verbeuren T. J. Van de Voorde J. Lameire N. H. Vanhoutte P. M. (2000). Endothelial Dysfunction in Diabetes. Br. J. Pharmacol. 130, 963–974. 10.1038/sj.bjp.0703393 10882379 PMC1572156

[B6] Falcone J. C. (1995). Endothelial Cell Calcium and Vascular Control. Med. Sci. Sports Exerc. 27, 1165–1169. 10.1249/00005768-199508000-00010 7476061

[B7] Filippini A. D’Amore A. D’Alessio A. (2019). Calcium Mobilization in Endothelial Cell Functions. Ijms 20, 4525. 10.3390/ijms20184525 PMC6769945 31547344

[B8] Filosa J. A. Yao X. Rath G. (2013). TRPV4 and the Regulation of Vascular Tone. J. Cardiovasc. Pharmacol. 61, 113–119. 10.1097/FJC.0b013e318279ba42 23107877 PMC3564998

[B9] Fitzgerald S. M. Kemp-Harper B. K. Parkington H. C. Head G. A. Evans R. G. (2007). Endothelial Dysfunction and Arterial Pressure Regulation during Early Diabetes in Mice: Roles for Nitric Oxide and Endothelium-Derived Hyperpolarizing Factor. Am. J. Physiology-Regulatory, Integr. Comp. Physiol. 293, R707–R713. 10.1152/ajpregu.00807.2006 17522117

[B10] Francis M. Qian X. Charbel C. Ledoux J. Parker J. C. Taylor M. S. (2012). Automated Region of Interest Analysis of Dynamic Ca2+ Signals in Image Sequences. Am. J. Physiology-Cell Physiol. 303, C236–C243. 10.1152/ajpcell.00016.2012 PMC3423022 22538238

[B11] Francis M. Waldrup J. R. Qian X. Solodushko V. Meriwether J. Taylor M. S. (2016). Functional Tuning of Intrinsic Endothelial Ca 2+ Dynamics in Swine Coronary Arteries. Circ. Res. 118, 1078–1090. 10.1161/circresaha.115.308141 26838791 PMC4818197

[B12] Gimbrone M. A. García-Cardeña G. (2016). Endothelial Cell Dysfunction and the Pathobiology of Atherosclerosis. Circ. Res. 118, 620–636. 10.1161/CIRCRESAHA.115.306301 26892962 PMC4762052

[B13] Hartmannsgruber V. Heyken W.-T. Kacik M. Kaistha A. Grgic I. Harteneck C. (2007). Arterial Response to Shear Stress Critically Depends on Endothelial TRPV4 Expression. PLoS ONE 2, e827. 10.1371/journal.pone.0000827 17786199 PMC1959246

[B14] Heathcote H. R. Lee M. D. Zhang X. Saunter C. D. Wilson C. McCarron J. G. (2019). Endothelial TRPV4 Channels Modulate Vascular Tone by Ca 2+ ‐induced Ca 2+ Release at Inositol 1,4,5‐trisphosphate Receptors. Br. J. Pharmacol., 14762. 10.1111/bph.14762 PMC6692577 31177523

[B15] Kansui Y. Garland C. J. Dora K. A. (2008). Enhanced Spontaneous Ca2+ Events in Endothelial Cells Reflect Signalling through Myoendothelial gap Junctions in Pressurized Mesenteric Arteries. Cell Calcium 44, 135–146. 10.1016/j.ceca.2007.11.012 18191200 PMC2635531

[B16] Ledoux J. Taylor M. S. Bonev A. D. Hannah R. M. Solodushko V. Shui B. (2008). Functional Architecture of Inositol 1,4,5-trisphosphate Signaling in Restricted Spaces of Myoendothelial Projections. Proc. Natl. Acad. Sci. U.S.A. 105, 9627–9632. 10.1073/pnas.0801963105 18621682 PMC2474537

[B17] Lin M. T. Jian M. Y. Taylor M. S. Cioffi D. L. Yap F. C. Liedtke W. (2015). Functional Coupling of TRPV4, Ik, and SK Channels Contributes to Ca 2+ ‐Dependent Endothelial Injury in Rodent Lung. Pulm. Circ. 5, 279–290. 10.1086/680166 26064452 PMC4449238

[B18] Liu L. Guo M. Lv X. Wang Z. Yang J. Li Y. (2021). Role of Transient Receptor Potential Vanilloid 4 in Vascular Function. Front. Mol. Biosci. 8, 677661. 10.3389/fmolb.2021.677661 33981725 PMC8107436

[B19] Ma X. Du J. Zhang P. Deng J. Liu J. Lam F. F.-Y. (2013). Functional Role of TRPV4-K Ca 2.3 Signaling in Vascular Endothelial Cells in Normal and Streptozotocin-Induced Diabetic Rats. Hypertension 62, 134–139. 10.1161/HYPERTENSIONAHA.113.01500 23648706

[B20] McFarland S. J. Weber D. S. Choi C.-s. Lin M. T. Taylor M. S. (2020). Ablation of Endothelial TRPV4 Channels Alters the Dynamic Ca2+ Signaling Profile in Mouse Carotid Arteries. Ijms 21, 2179. 10.3390/ijms21062179 PMC7139994 32235694

[B21] Mendoza S. A. Fang J. Gutterman D. D. Wilcox D. A. Bubolz A. H. Li R. (2010). TRPV4-mediated Endothelial Ca2+ Influx and Vasodilation in Response to Shear Stress. Am. J. Physiology-Heart Circulatory Physiol. 298, H466–H476. 10.1152/ajpheart.00854.2009 PMC2822567 19966050

[B22] Monaghan K. McNaughten J. McGahon M. K. Kelly C. Kyle D. Yong P. H. (2015). Hyperglycemia and Diabetes Downregulate the Functional Expression of TRPV4 Channels in Retinal Microvascular Endothelium. PLOS ONE 10, e0128359. 10.1371/journal.pone.0128359 26047504 PMC4457535

[B23] Nishijima Y. Zheng X. Lund H. Suzuki M. Mattson D. L. Zhang D. X. (2014). Characterization of Blood Pressure and Endothelial Function in TRPV4-Deficient Mice with L -NAME- and Angiotensin II-Induced Hypertension. Physiol. Rep. 2, e00199. 10.1002/phy2.199 24744878 PMC3967682

[B24] Park Y. Capobianco S. Gao X. Falck J. R. Dellsperger K. C. Zhang C. (2008). Role of EDHF in Type 2 Diabetes-Induced Endothelial Dysfunction. Am. J. Physiology-Heart Circulatory Physiol. 295, H1982–H1988. 10.1152/ajpheart.01261.2007 PMC2614585 18790831

[B25] Qian X. Francis M. Köhler R. Solodushko V. Lin M. Taylor M. S. (2014). Positive Feedback Regulation of Agonist-Stimulated Endothelial Ca 2+ Dynamics by K Ca 3.1 Channels in Mouse Mesenteric Arteries. Arterioscler Thromb. Vasc. Biol. 34, 127–135. 10.1161/ATVBAHA.113.302506 24177326 PMC4181598

[B26] Qian X. Francis M. Solodushko V. Earley S. Taylor M. S. (2013). Recruitment of Dynamic Endothelial Ca2+Signals by the TRPA1 Channel Activator AITC in Rat Cerebral Arteries. Microcirculation 20, 138–148. 10.1111/micc.12004 22928941 PMC3524345

[B27] Rajan S. Schremmer C. Weber J. Alt P. Geiger F. Dietrich A. (2021). Ca2+ Signaling by TRPV4 Channels in Respiratory Function and Disease. Cells 10, 822. 10.3390/cells10040822 33917551 PMC8067475

[B28] Sonkusare S. K. Bonev A. D. Ledoux J. Liedtke W. Kotlikoff M. I. Heppner T. J. (2012). Elementary Ca 2+ Signals through Endothelial TRPV4 Channels Regulate Vascular Function. Science 336, 597–601. 10.1126/science.1216283 22556255 PMC3715993

[B29] Sullivan M. N. Earley S. (2013). TRP Channel Ca2+ Sparklets: Fundamental Signals Underlying Endothelium-dependent Hyperpolarization. Am. J. Physiology-Cell Physiol. 305, C999–C1008. 10.1152/ajpcell.00273.2013 PMC3840200 24025865

[B30] Sullivan M. N. Francis M. Pitts N. L. Taylor M. S. Earley S. (2012). Optical Recording Reveals Novel Properties of GSK1016790A-Induced Vanilloid Transient Receptor Potential Channel TRPV4 Activity in Primary Human Endothelial Cells. Mol. Pharmacol. 82, 464–472. 10.1124/mol.112.078584 22689561 PMC3422704

[B31] Taylor M. S. Choi C.-s. Bayazid L. Glosemeyer K. E. Baker C. C. P. Weber D. S. (2017). Changes in Vascular Reactivity and Endothelial Ca2+dynamics with Chronic Low Flow. Microcirculation 24, e12354. 10.1111/micc.12354 PMC5404954 28106317

[B32] Wilson C. Zhang X. Lee M. D. MacDonald M. Heathcote H. R. Alorfi N. M. N. (2020). Disrupted Endothelial Cell Heterogeneity and Network Organization Impair Vascular Function in Prediabetic Obesity. Metabolism 111, 154340. 10.1016/j.metabol.2020.154340 32791171 PMC7538703

[B33] Wolf M. P. Hunziker P. (2020). Atherosclerosis: Insights into Vascular Pathobiology and Outlook to Novel Treatments. J. Cardiovasc. Trans. Res. 13, 744–757. 10.1007/s12265-020-09961-y 32072564

[B34] Zhang P. Sun C. Li H. Tang C. Kan H. Yang Z. (2018). TRPV4 (Transient Receptor Potential Vanilloid 4) Mediates Endothelium-dependent Contractions in the Aortas of Hypertensive Mice. Hypertension 71, 134–142. 10.1161/HYPERTENSIONAHA.117.09767 29109190

